# Utility of non-HDL-C in predicting proteinuria remission of idiopathic membranous nephropathy: a retrospective cohort study

**DOI:** 10.1186/s12944-021-01558-x

**Published:** 2021-09-29

**Authors:** Lei Dong, Wang Wei, Min Han, Gang Xu

**Affiliations:** 1 Department of Nephrology, Tongji Hospital, Tongji Medical College, Huazhong University of Science and Technology, No. 1095 Jiefang Avenue, Wuhan, D-430030 Hubei China; 2 Tongji Hospital, Tongji Medical College, Huazhong University of Science and Technology, No. 1095 Jiefang Avenue, Wuhan, D-430030 Hubei China

**Keywords:** Non-high-density lipoprotein cholesterol, Idiopathic membranous nephropathy, Remission, Total cholesterol

## Abstract

**Background:**

Idiopathic membranous nephropathy (IMN) may have various clinical outcomes. Hyperlipidemia is quite common in IMN. However, the utility of the lipid profile in predicting outcomes remains unknown. This study aimed to explore the correlation between hyperlipidemia and proteinuria remission in IMN.

**Methods:**

256 patients who diagnosed with IMN confirmed by renal biopsy in Wuhan Tongji Hospital from January 2016 to October 2020 were included in this study. The end point was defined as a combination of partial and complete remission. Cox proportional-hazards regression analysis and Kaplan–Meier curve were applied to assess the prognostic value of the lipid profile for proteinuria remission.

**Results:**

A total of 153 (59.8%) patients achieved remission and 103 (40.2%) did not. The levels of total cholesterol, low-density lipoprotein, and non-high-density lipoprotein were significantly lower in the remission group than in the non-remission group. Non-high-density lipoprotein level revealed the strongest correlation with proteinuria (Spearman’s rho = 0.42; *P* < 0.001). The multivariate analysis demonstrated that serum total cholesterol [hazard ratio (HR): 0.883; 95% confidence interval (CI): 0.813–0.958; *P* = 0.003] and non-high-density lipoprotein cholesterol (HR: 0.892; 95% CI: 0.820–0.970; *P* = 0.007) levels were independent markers to predict proteinuria remission in IMN.

**Conclusions:**

Among the lipid profile, the non-high-density lipoprotein level exhibited the strongest correlation with proteinuria in IMN. Moreover, elevated serum cholesterol and non-high-density lipoprotein cholesterol concentrations at baseline predicted probability of proteinuria non-remission in IMN.

## Introduction

Idiopathic membranous nephropathy (IMN) accounts for one of the most common primary glomerulonephritis and exhibits long natural course [1, 2]. Its incidence in China has increased dramatically in recent years, especially in elderly patients [2], which may have partly resulted from exposure to high levels of particulate matter of < 2.5 μm over long periods [3]. IMN, considered as an immune-complex-mediated disease, often manifests as a nephrotic syndrome (NS) with hyperlipidemia (hypercholesterolemia and hypertriglyceridemia) [1].

Our understanding of its pathophysiological mechanism has recently gained more momentum after identifying M-type phospholipase A_2_ receptor (PLA2R) as the major antigenic target [4, 5]. A total of 40–50% of patients achieve spontaneous remission while the remaining get deterioration of kidney function, step to the end stage of kidney disease in 5 to 10 years [6]. The clinical variables, including older age at onset, male sex, persisting hypertension, hyperlipidemia and/or hypoalbuminemia, nephrotic-range proteinuria, high PLA2R titer, and pathological variables, including fibrosis of tubule interstitium, and focal segmental sclerosis (FSGS), were considered as the risk factors for kidney function progression in previous studies [6–9].

The linkage between hypercholesterolemia and outcomes of primary glomerular diseases has been reported [10]. In a multicenter cohort study involving 761 children, 72% of participants not in remission had hypercholesterolemia than 43% of those in remission (*P* < 0.001), indicating an association between hypercholesterolemia and not achieving remission [10]. The effect of lipid profile parameters on IMN has been rarely studied.

In patients with chronic kidney disease, the traditional lipid profile [triglyceride (TG), total cholesterol (TC), high-density lipoprotein cholesterol (HDL-C), and low-density lipoprotein cholesterol (LDL-C)] was commonly evaluated as a risk factor for cardiovascular disease [11]. Serum non-high-density lipoprotein cholesterol (non-HDL-C), is a new parameter, presented as the difference between TC and HDL-C. It is the sum of serum LDL-C, intermediate-density lipoprotein-C (IDL-C), and very-LDL-C levels (VLDL-C) [12]. It has been reported that serum non-HDL-C is a critical predictor for cardiovascular disease, maybe even better than LDL-C [13, 14]. Compared with traditional lipid parameters, non-HDL-C was considered more relevant with albuminuria in type 1 as well as type 2 diabetes, indicating its potential role in the pathogenesis of diabetic nephropathy [15, 16]. To our knowledge, the role of non-HDL-C and other lipid parameters in the outcome of proteinuria in IMN has been rarely investigated.

This study used the cohort in our center to assess the utility of serum lipid parameters in predicting proteinuria remission in IMN.

## Methods

### Study design and patients

This retrospective study included all consecutive patients with IMN confirmed by biopsy from January 01, 2016, to October 31, 2020, at Tongji Hospital in Wuhan, China. MN was pathologically diagnosed by thickening of capillary walls, and subepithelial depostion of immune globulin G (IgG) and complement (C) 3 alongside capillary walls [17]. Inclusion criteria: (i) patients without previous treatment of steroid and immunosuppressants (6 months before screening); (ii) the follow-up time longer than 6 months; (iii) age ≥ 18 years; and (iv) no use of lipid-lowering medication upon admission. Exclusion criteria: patients with secondary MN caused by tumors, autoimmune disease, metabolic disease, hepatitis B virus–related disease and the interval between baseline measurements and kidney biopsy exceeding 3 years.

This study complied with the Helsinki declaration. Informed consent was waived by the ethics review board of Tongji Hospital, Tongji Medical College, Huazhong University of Science and Technology (No. TJ-IRB20210701).

### Data collection

Data on general demographics (age and sex) and anthropometric measurements [weight, height, and systolic/diastolic blood pressure (BP)] were collected from electronic medical records. Blood samples for biochemical parameters of all patients were taken on the same day of their first admission, before a renal biopsy. The laboratory data included serum creatinine (SCr), urine protein, albumin and lipid parameters. The lipid profile parameters consist of TC, TG, HDL-C, and LDL-C were all measured by enzyme colorimetry. HDL-C was subtracted from TC to calculate the value of Non-HDL-C. Pathological parameters included lesions of FSGS, crescent formation, tubular atrophy, Ehrenreich–Churg morphological stage and immunohistological staining which included IgG subgroup, immune globulin A (IgA), immue globulin M (IgM), C3, C1q, and PLA2R. Tubular (T) atrophy: T0 was defined as absent, T1 was mild (< 25%), T2 was moderate (25–50%) and T3 was severe (> 50%) [18].

### Therapy and definitions

Patients received supportive and immunosuppressive therapy depending on their degree of proteinuria, kidney function, observational period, and so forth, based on the Kidney Disease: Improving Global Outcomes (KDIGO) guidelines [19, 20]. Supportive therapy included anticoagulant, renin–angiotensin system (RAS) blockade, other antihypertensive agents, and diuretics. Immunosuppressive treatment included corticosteroids, cyclophosphamide, tacrolimus, mycophenolate mofetil, and rituximab. Patients with complete remission should meet with a minor proteinuria (< 0.3 g/d) accompanied with normal concentration of serum albumin and creatinine. Patients with partial remission should meet with moderate proteinuria (< 3.5 g/d) and a 50% or greater reduction of proteinuria, accompanied with stable creatinine concentration and improved or normalized albumin value [19]. Both complete and partial remissions were considered remission.

### Statistical analysis

Software SPSS (version 24.0, IBM, US) was applied for statistical analyses. None of the continuous variables were normally distributed, according to the results of normality tests. For non-normally distributed continuous variables, data were presented as median (interquartile range, IQR); for categorical variables, data were presented as numbers and proportions. Mann–Whitney test was applied in non-normally distributed variables for univariate comparisons, while *χ*^2^ test or Fisher exact test were applied in categorical variables for univariate comparisons, as appropriate. The associations between lipid parameters and proteinuria and serum albumin values were compared using Spearman’s rho. The end point was defined as the remission of proteinuria. Time-to-event analyses were performed using Cox regression analyses. The models included the risk factors of interest and were adjusted for the covariates with a *P* value <.1 when performed in the univariate analysis or with clinical significance, that is, sex, age, body mass index (BMI), BP, SCr, albumin, and FSGS. The log-rank test was used for comparing proteinuria remission in paticipants with above and below the median of TC and non-HDL-C levels. A significant difference was defined as a *P* value <0.05.

## Results

### Cohort description

**Fig. 1:**
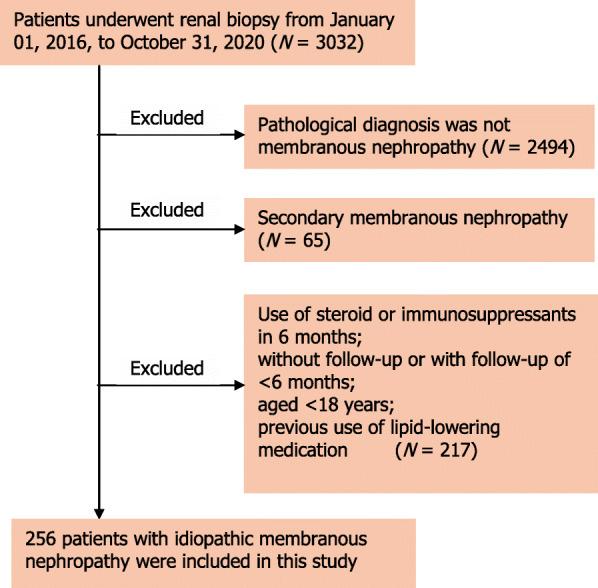
Flow chart of participant selection in this study. From the total 3032 patients in our single center, we screened patients with idiopathic membranous nephropathy who met the inclusion criteria. A total of 256 participants were included

After applying the exclusion criteria, this study cohort included a total of 256 participants (Fig. 1). The baseline characteristics of this cohort was summarized in Table 1. All patients were of Han nationality. In the remission group, participants exhibited higher systolic and diastolic BP, higher albumin level, and lower TC, non-HDL-C, and LDL-C levels at disease diagnosis (Table 1). In the use of RAS blockade and steroid and immunosuppressant, no significant difference was noted between the two groups (Table 1).

**Table 1:** Baseline characteristics according to outcome

Characteristic	All patients (*N* = 256)	Without remission (*N* = 103)	Remission (*N* = 153)	*P* value
Demographics				
Age, year	49 (40–56)	50 (40–57)	49 (38–56)	0.276
Male sex, no. (%)	103 (40.2)	36 (34.9)	67 (43.7)	0.157
Ethnicity				
Asian	256 (100)	103 (100)	153 (100)	> 0.99
Medical history				
Hypertension, no. (%)	46 (18)	16 (16.5)	30 (19.6)	0.405
Diabetes, no. (%)	17 (6.6)	9 (8.7)	8 (5.2)	0.269
Anthropometric measurements				
Systolic BP, mm Hg	130 (118–140)	126 (116–138)	130 (120–144)	0.008^**^
Diastolic BP, mm Hg	82 (75–90)	79 (75–87)	83 (76–92)	0.026^*^
BMI, kg/m^2^	24.2 (22.4–27.1)	24.2 (21.7–26.8)	24.3 (22.4–27.3)	0.496
Kidney function measurements				
Serum creatinine, μmol/L	79 (63–99)	83 (63–100)	77 (62–99)	0.606
Urine protein, g/d	4.3 (2.3–7.2)	3.8 (2.3–6.2)	4.5 (2.7–7.6)	0.086
Albumin, g/L	26.9 (22.6–32.7)	26.7 (21.9–30.7)	27.8 (24.1–33.9)	0.047^*^
Plasma lipid levels				
TC, mmol/L	6.59 (5.25–8.19)	7.13 (5.56–8.49)	6.20 (4.99–7.89)	0.011^*^
Non-HDL-C, mmol/L	5.12 (3.90–6.76)	5.84 (4.13–7.17)	4.92 (3.73–6.43)	0.027^*^
HDL-C, mmol/L	1.28 (1.04–1.63)	1.29 (1.05–1.62)	1.28 (1.04–1.63)	0.927
LDL-C, mmol/L	3.73 (2.64–5.20)	4.01 (2.96–5.87)	3.49 (2.55–5.00)	0.02^*^
Triglyceride, mmol/L Medications	2.23 (1.49–3.59)	2.18 (1.56–3.27)	2.28 (1.41–3.66)	0.926
RAS blockade, no. (%)	242 (94.5)	96 (93.2%)	146 (95.4)	0.577
Steroid and immunosuppressant, no. (%)	173 (67.6)	65 (63.1)	108 (70.6)	0.210

^*^P < 0.05; ^**^P < 0.01

Table 2 shows a comparison of pathological characteristics in different groups. No significant differences were noted in morphological staging, crescent formation, tubular atrophy, and deposition of IgG4, IgA, IgM, C3, C1q, and PLA2R. The remission group had a higher proportion of superimposed FSGS compared with the group without remission (Table 2). However, in the Cox proportional-hazards model, the univariate analysis indicated that FSGS was not a predictor for proteinuria remission (HR: 0.881; 95% CI: 0.555–1.400; *P* = 0.592; data not shown).

**Table 2:** Comparison of pathological characteristics between groups

	All patients (*N* = 256)	Without remission (*N* = 103)	Remission (*N* = 153)	*P* value
Morphological staging, no.(%)				0.108
I+ II	232 (90.6)	85 (82.6)	147 (96.1)	
III + IV	24 (9.4)	18 (17.5)	6 (3.9)	
Lesions of FSGS, no. (%)	28 (10.9)	6 (5.8)	22 (14.4)	0.032^*^
Crescent formation, no. (%)	34 (13.3)	16 (15.5)	18 (11.8)	0.384
Tubular atrophy, no. (%)				0.382
T0	31 (12.1)	17 (16.5)	14 (9.2)	
T1	178 (69.5)	59 (57.3)	119 (77.8)	
T2	43 (16.8)	26 (25.2)	17 (11.1)	
T3	4 (1.6)	1 (1.0)	3 (2)	
IgG4-dominant deposition, no. (%)	182 (71.1)	74 (71.8)	108 (70.6)	0.828
IgA deposition, no. (%)	39 (15.2)	19 (18.4)	20 (13.1)	0.241
IgM deposition, no. (%)	66 (25.8)	31 (30.1)	35 (22.9)	0.195
C3 deposition, no. (%)	222 (86.7)	88 (85.4)	134 (87.6)	0.620
C1q deposition, no. (%)	86 (33.6)	33 (32)	53 (34.6)	0.666
PLA2R deposition, no. (%)	199 (77.7)	76 (73.8)	123 (80.4)	0.213

^*^P < 0.05

### Correlations between serum lipid levels and proteinuria and serum albumin levels

Significant positive correlations were found between TC, non-HDL-C, LDL-C, and TG levels and proteinuria (Table 3), while inverse correlations were found between these four parameters and serum albumin concentrations (Table 3). Inverse correlation was found between the HDL-C level with proteinuria (Table 3) rather than serum albumin (Table 3). Among the serum lipids, non-HDL-C exhibited the strongest correlation with proteinuria and the serum albumin level.

**Table 3:** Correlations between lipid profile parameters and proteinuria and serum albumin levels

	Proteinuria		Serum albumin	
	rs	*P*	rs	*P*
TC	0.351	< 0.001^**^	−0.419	< 0.001^**^
HDL-C	−0.198	0.002^**^	0.044	0.495
Non-HDL-C	0.422	< 0.001^**^	−0.435	< 0.001^**^
LDL-C	0.288	< 0.001^**^	− 0.38	< 0.001^**^
TG	0.378	< 0.001^**^	−0.15	0.018^*^

*Rs* Spearman’s rank correlation coefficient

^*^P < 0.05; ^**^P < 0.01

### Adjusted association of serum lipid levels with clinical outcomes

The HRs and 95% CIs for remission of IMN from each dyslipidemia type are presented in Table 4. TC and non-HDL-C were implied as risk factors for persistant proteinuria in IMN by the univariate Cox regression analysis in model 1. After adjusting for general variables including sex, age, BMI, and BP in model 2, TC and non-HDL-C remained as risk factors. After further adjusting all variables in model 2 plus SCr, serum albumin, and FSGS, elevated TC and non-HDL-C concentrations were indicated as independent risk factors for non-remission of proteinuria in model 3.

**Table 4:** Adjusted hazard ratios (HR) with 95% confidence intervals (CI) for the remission of idiopathic membranous nephropathy

	Model 1	*P*	Model 2	*P*	Model 3	*P*
	HR (95% CI)		HR (95% CI)		HR (95% CI)	
TC	0.908 (0.842–0.980)	0.013^*^	0.881 (0.813–0.956)	0.002^**^	0.883 (0.813–0.958)	0.003^**^
HDL-C	–	0.56	–	–	–	–
Non-HDL-C	0.914 (0.845–0.988)	0.024^*^	0.888 (0.817–0.965)	0.005^**^	0.892 (0.820–0.970)	0.007^**^
LDL-C	–	0.139	–	–	–	–
TG	–	0.371	–	–	–	–

^*^P < 0.05; ^**^P < 0.01

Model 1 was an unadjusted model

Model 2 was adjusted for sex, age, BMI, and BP

Model 3 was adjusted for all the variables in model 2 plus SCr, albumin, and FSGS

**Fig. 2:**
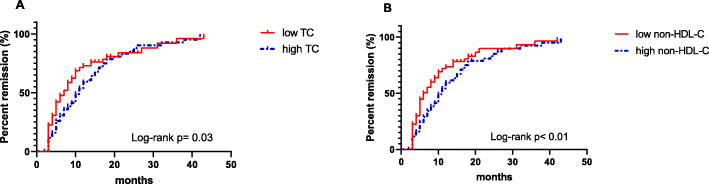
Kaplan-Meier curve for proteinuria remission according to the serum TC and non-HDL-C levels

Based on the median values, TC and non-HDL-C were divided into high- and low-level groups. A higher probablility of remission was achieved in patients wtih low-TC rather than high-TC (log-rank test, *P* = 0.03, Fig. 2A) when applied in Kaplan–Meier analysis. Similar results were noted for non-HDL-C (log-rank test, *P* < 0.01, Fig. 2B).

## Discussion

This study examined the relationship between lipid profile and proteinuria remission in patients with biopsy-confirmed IMN. Our findings confirmed that hypercholesterolemia and hypertriglyceridemia paralleled the severity of proteinuria in IMN. Elevated serum TC and non-HDL-C levels at the initial visit were independent risk factors for persistent proteinuria in IMN, indicating the importance of screening these risk factors on admission.

In this study, baseline systolic BP and diastolic BP were mildly higher in the remission group than in the non-remission group. This finding could possibly be explained by a higher proportion of patients with a history of hypertension in the remission group (19.6%) than in the non-remission group (16.5%). Patients with elevated BP might receive a higher dose of RAS blockade, i.e. perindopril 8 mg/d or irbesartan 150 mg/d. But patients with normal BP only received half-dose of RAS blockade, i.e. perindopril 4 mg/d or irbesartan 75 mg/d, or even no use of it, if intolerable. Fan Fan Hou et al. found that uptitration of RAS blockade achieved a greater reduction in proteinuria compared with their conventional dosages in the ROAD study [21]. So it is reasonable that in our study participants with high BP at baseline who received higher dosage of RAS blockade were prone to achieve proteinuria remission. To find out whether or not the use of RAS blockade will affect the associations between lipids and proteinuria remission, we performed an additional analysis with multivariable Cox model to rule out the effect of RAS blockade, and the result showed that RAS blockade did not alter the significant associations (data not shown).

An FSGS lesion has been considered to be an indicator for poor outcome of kidney function in some studies [22–24]. However, when proteinuria remission was set as the end point, other studies and the present study did not find a significant difference between patients with IMN with and without FSGS [25]. It suggested that superimposed FSGS in IMN might predict a poor prognosis of renal function but could not predict the prognosis of proteinuria. However, the association between proteinuria outcomes and FSGS lesion subgroups was not performed in the present study due to the limited number of cases.

It is acknowledged that the metabolic disorders of lipid and lipoprotein in NS parallel the severity of proteinuria and contribute to the progression of renal function [26]. Consistent with it, we confirmed that hypercholesterolemia and hypertriglyceridemia were correlated with the severity of proteinuria in IMN. However, the lipid alterations in different renal pathological types might differ. Hypercholesterolemia was the most frequent complication in IMN (82%) among four common primary glomerular diseases including membranous nephropathy, IgA nephropathy, minimal change disease and FSGS [10]. In addition, our result indicated non-HDL-C as the most relevant marker among other markers in the lipid profile with proteinuria in IMN.

The role of hyperlipidemia in outcomes of primary glomerular diseases has been rarely investigated. As mentioned earlier, an association between hypercholesterolemia and not achieving remission in primary glomerulonephritis has been implied. In patients with IgA nephropathy and global glomerulosclerosis, hypertriglyceridemia predicted poor outcome of kidney disease [27]. Moreover, our multivariable analysis result inferred that elevated TC and non-HDL-C levels independently predicted non-remission of proteinuria in IMN. Besides lipid parameters, non-metabolic parameters such as decreased hemoglobin levels have also been observed to be relevant with the progression of albuminuria and mortality [28, 29].

Proteinuria can result in hyperlipidemia, mediated by lipids and lipoproteins metabolic pathways [26, 30]. In turn, hyperlipidemia may contribute to IMN pathogenesis through key genes, including apolipoprotein A1, apolipoprotein B, apolipoprotein C3, cholesteryl ester transfer protein, and phospholipase A2 group XIIB, as reported in a genomics study using renal cortex tissue from patients with IMN and healthy controls [31]. However, the molecular basis between hyperlipidemia and proteinuria in NS largely remains to be elucidated. Only the link between hypertriglyceridemia and proteinuria, which was mediated by increasing circulating angiopoietin-like 4 levels, has been elucidated [32, 33]. The molecular relationship between hypercholesterolemia and proteinuria remains unclear [33]. Further researches are required to focus on the molecular mechanism of hyperlipidemia in the pathogenesis of IMN.

### Study strength and limitations

This study demonstrated the predictive role of TC and non-HDL-C in proteinuria remission in IMN, strengthening the idea that lipid monitoring should be considered in assessing the risk of IMN. However, several weaknesses limited the strength of this article. As a retrospective observational study, a bias might exist in the result analysis; our data did not clarify any threshold for treatment. Additionally, due to the lack of longitudinal follow-up data of lipid levels, we did not know how lipid management affected proteinuria remission in IMN. Future studies are necessary to examine the relationship between longitudinal lipid levels and clinical outcomes. We could not determine whether lipid levels were measured in the fasting or non-fasting state, which might have interfered with the accuracy of TG. However, a non-fasting status did not affect the measuement of TC and non-HDL-C [10, 34]. Therefore, clinicians should focus on these two applicable markers and sequentially reduce proteinuria to delay the progression of IMN.

## Conclusions

In this longitudinal study of patients with IMN, the magnitude of hyperlipidemia paralleled the severity of proteinuria. Lower levels of serum TC and non-HDL-C at onset were independently associated with a higher remission rate of proteinuria, suggesting a potential role of TC and non-HDL-C in the pathogenesis of IMN. The result highlighted the need for screening dyslipidemia in the early stage in patients with IMN, who needed more intensive follow-up and perhaps lipid-lowering interventions.

## Data Availability

The datasets generated and/or analyzed in the present study are not publicly available due to individual privacy but are available from the corresponding author on reasonable request.

## Abbreviations

BMI: Body mass index
BP: Blood pressure
C3: Complement 3
FSGS: Focal segmental sclerosis
HDL-C: High-density lipoprotein
Ig: Immune globulin
IMN: Idiopathic membranous nephropathy
LDL-C: Low-density lipoprotein
non-HDL-C: Non-high-density lipoprotein cholesterol
NS: Nephrotic syndrome
PLA2R: M-type phospholipase A_2_ receptor
SCr: Serum creatinine
TC: Total cholesterol
TG: Triglyceride
